# Survival and Incidence of Gastric Neuroendocrine Tumors: A Surveillance, Epidemiology, and End Results (SEER) Database Analysis

**DOI:** 10.7759/cureus.104120

**Published:** 2026-02-23

**Authors:** Grace S Saglimbeni, Tyson J Morris, Laura M Cogua, Connor J Tupper, Peter T Silberstein

**Affiliations:** 1 Medicine, Creighton University School of Medicine, Phoenix, USA; 2 Orthopedic Surgery, Zucker School of Medicine at Hofstra/Northwell, New Hyde Park, USA; 3 Hematology and Oncology, Creighton University School of Medicine, Omaha, USA

**Keywords:** demographics, gastric neuroendocrine tumors, gnet, health disparities, incidence, prognostic factors, seer database, stage at diagnosis, survival analysis, tumor size

## Abstract

Introduction: Gastric neuroendocrine tumors (GNETs) are slow-growing tumors derived from enterochromaffin-like cells whose prognosis depends on the type. Prior GNET studies have shown an increasing incidence, but survival analyses have been more limited. This study aims to investigate if the increasing incidence trend continues and better describe factors associated with survival for GNET patients.

Methods: In this retrospective population-based cohort study, patients diagnosed with GNET between 2000 and 2020 were selected from the Surveillance, Epidemiology, and End Results (SEER) database. Additional variables collected were age, sex, race, stage, presence of metastases, tumor size, treatment status for surgery, radiation, and chemotherapy, median household income, and population size. Descriptive statistics, population-based incidence, and Cox regression analyses were performed.

Results: A total of 6512 patients were included. The one- and five-year survival rates were 90.4% and 83.8%, respectively. The population-adjusted incidence ranged from 0.272/100,000 in 2000 to 0.680/100,000 in 2020. The total percent change in incidence over the study range was 104.1% with an annual percent change of 4.27%, which met significance (p<0.05). Chi-square analyses demonstrated significantly higher 1- and 5-year cancer-specific survival among patients aged <50 years, males, Whites, those with localized stage, no metastases, tumor size 0.1-2.0 cm, and no receipt of surgery or chemotherapy (all p < 0.001). Cox regression results showed that age of 70+ years, regional and distant staging, any distant metastases, and tumor size >2.0cm and >5.0cm were associated with shorter survival (p<0.05). Additionally, females, Hispanic patients, and recipients of surgery were associated with longer survival (p<0.05).

Significance: The findings show GNET incidence has continued to increase over the past two decades. Additionally, clinical factors including stage, extent of metastasis, tumor size and socioeconomic factors like age, gender, and race were associated with changes in GNET survival. In the context of the increasing incidence of GNET, these findings describe factors associated with lower- and higher-risk tumors. Further assessment of these risk factors can benefit future research to better understand why GNET incidence is increasing, aid in risk stratification of GNET patients, and improve the prognosis of GNET.

## Introduction

Gastric neuroendocrine tumors (GNETs) are rare, slow-growing neoplasms that arise from enterochromaffin-like cells of the stomach [1]. They represent a heterogeneous group of tumors that are classified into three subtypes that differ in behavior and prognosis. Type I tumors, which account for 70-80% of cases, are typically associated with chronic atrophic gastritis, whereas type II tumors, comprising about 5% of cases, develop secondary to gastrinomas or multiple endocrine neoplasia type 1 (MEN1). In contrast, type III tumors represent 20-30% of cases and are generally diagnosed in the absence of hypergastrinemia [1,2].

Although GNETs are relatively uncommon, comprising only 0.1-0.6% of all gastric cancers [2], their incidence has grown substantially in recent decades. Previous Surveillance, Epidemiology, and End Results (SEER) studies reported an increase from 0.31 cases per 1,000,000 individuals in 1975 to 4.85 per 1,000,000 in 2014 [3], with subsequent analyses showing a further increase to 6.149 cases per 1,000,000 individuals by 2016 [4]. More recent United States studies indicate that the overall rise in non-cardia gastric cancer is largely driven by GNETs [5], and international studies corroborate similar upward trends across other populations [6,7]. The observed rise in incidence likely reflects improved detection through advances in endoscopy and immunohistochemistry, and more complete registry reporting, leading to recognition of cases that may have previously gone undiagnosed [8].

Beyond incidence, outcomes for GNET patients vary by subtype and additional prognostic factors. Type I tumors are generally indolent with low metastatic potential, type II tumors demonstrate intermediate behavior, and type III tumors are more aggressive, often presenting with nodal or hepatic metastases and poorer survival [1,2]. Consistent with their biology, Type I and II GNETs are commonly managed with endoscopic resection, whereas Type III tumors typically require surgical resection with lymphadenectomy. Outcomes are further shaped by tumor characteristics, with size, histology, and grade, including the Ki-67 labeling index, recognized as important for risk stratification [9,10]. Stage, however, remains the strongest determinant of outcome as distant disease confers more than a tenfold increase in mortality compared with localized tumors [11].

Despite these insights, significant gaps in our knowledge of GNETs remain. While multiple prior analyses have documented the increasing incidence of GNETs, survival analyses are comparatively limited, and few investigations have comprehensively assessed the clinical and demographic factors that may influence prognosis. To address these gaps, our study utilizes population-based SEER data with two primary objectives: (1) to evaluate whether the increasing incidence of GNETs has persisted in the contemporary period (2000-2020), and (2) to assess cancer-specific survival in a modern U.S. cohort. As secondary objectives, we explored associations between clinical and sociodemographic factors and survival outcomes.

The contents of this article were previously presented as a poster at the Association of VA Hematology/Oncology 2024 meeting in Atlanta, Georgia, on September 21, 2024.

## Materials and methods

Using the SEER database (SEER 17 registry grouping) [12], GNET cases diagnosed between 2000 and 2020 using histology codes 8246 and 8249 were selected (SEER accessed July 2024) in this retrospective population-based cohort study. The SEER 17 registries comprise population-based cancer registries across multiple U.S. geographic regions and, based on U.S. Census estimates, cover approximately 26.5% of the U.S. population, supporting broad representativeness of cancer incidence and survival patterns within participating areas [13]. Follow-up time was determined using SEER survival data through the end of the study period; no minimum follow-up duration was required for inclusion. Patients were not excluded based on cause of death; non-cancer-specific deaths were treated as censored observations in the calculation of cancer-specific survival. All cases had a minimum follow-up of one year. Patients were excluded if the cause of death was not categorized as cancer-specific death. Cancer-specific survival (CSS) was tracked through 60 months after diagnosis. Patients were not excluded based on survival duration, and deaths occurring within one year of diagnosis were included in the analysis. Variables analyzed included age, gender, race, stage at diagnosis, tumor size, presence of metastasis, surgical status, radiation status, chemotherapy status, time from diagnosis to treatment, median household income of home county, and population size of the home county.

Age groups included less than 50 years, 50-69 years, or 70 years or older. Gender was male and female. Race was Non-Hispanic White, Non-Hispanic Black, Hispanic, Asian, or American Indian/Alaska Native. The SEER-based combined staging used designations of localized, regional, or distant for the stage at diagnosis. Tumor size categories (0.1-2.0 cm, 2.1-5.0 cm, and >5.0 cm) were selected based on clinically relevant thresholds reported in prior literature, where lesions >2 cm have been associated with increased metastatic risk and adverse outcomes [14]. Presence of metastasis and treatment variables (surgery, radiation, and chemotherapy) were coded dichotomously as yes versus no/unknown, consistent with SEER reporting structure. Cases coded as “unknown” were grouped with “no” to preserve cohort size; however, we acknowledge that this approach may introduce exposure misclassification. Time from diagnosis to treatment groups was within one month, between one and two months, and over two months. The median household income in the patient's county of residence, as reported by United States census data, was under $50,000, $50,000-$74,999, and $75,000 or higher. Income data were derived from SEER-linked U.S. Census county-level estimates. The population size of the patient's residential area was classified as either metropolitan or non-metropolitan.

Incidence was determined using the SEER*Stat (Version 8.4.3) according to United States census data [12]. Descriptive statistics were completed for the whole cohort. One- and five-year CSS proportions were compared across covariate groups using chi-square tests. Missing data were handled using complete-case analysis, with “unknown” categories retained where applicable. Variables included in the multivariable Cox proportional hazards model were selected based on clinical relevance and prior literature. The proportional hazards assumption was evaluated using graphical methods. Cox proportional hazard analyses determined associations between covariates and survival. The significance for all analyses was set at alpha=0.05. SPSS Version 29 (IBM Corp., Armonk, NY, USA) was used for all statistical analyses. SEER*Stat case selection criteria are available upon reasonable request.

## Results

**Figure 1:**
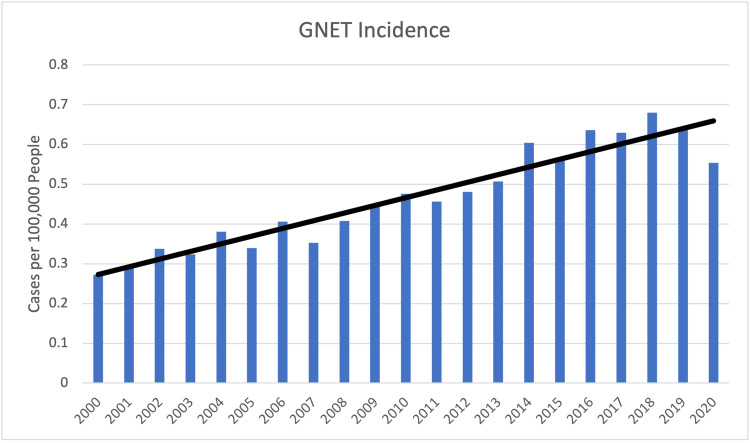
Average incidence of GNET between 2000 and 2020. GNET: Gastric neuroendocrine tumors

A total of 6,512 eligible GNET cases were identified in the SEER database during the study period after applying histologic and inclusion criteria. The population-adjusted incidence ranged from 0.272/100,000 in 2000 to 0.680/100,000 in 2020. The total percent change in incidence over the study range was 104.1% with an annual percent change of 4.27%, which met significance (p<0.05) (Figure 1).

The majority of included patients were female (3,910/6,512, 60.0%). The majority were White (n=3,606/6,512, 55.4%), followed by Hispanic (n=1,499/6,512, 23.0%), and Blacks (n=834/6,512, 12.8%). Most patients had localized staging (n=3,713/6,512, 57.0%) followed by distant (n=733/6,512, 11.3%). The majority had tumors measuring 0.1-2.0cm (n=2,384/6512, 36.6%). Most underwent surgical intervention (n=3,723/6,512, 57.2%) but did not receive radiation (n=6,295/6,512, 96.7%) or chemotherapy (n=5,854/6,512, 89.9%). Treatment timing relative to diagnosis showed that most were treated over two months after diagnosis (n=3,382/6,512, 51.9%). Complete descriptive statistics are presented in Table 1.

The one and five-year survival rates were 90.4% and 83.8%, respectively. Chi-square analysis demonstrated that males, patients younger than 50 years old, Whites, patients with localized tumors, patients with no metastasis, those who have tumor sizes 0.1cm-2.0cm, and those with no receipt of surgery or chemotherapy were all significantly associated with increased both one- and five-year survival (p’s<0.001). No significant relationships were found between one- or five-year survival and median household income or population size. Complete chi-square comparisons are presented in Table 1.

**Table 1:** Patient descriptive statistics and one- and five-year survival following a diagnosis of GNET. *Some category totals may not sum to N due to missing or incomplete data.

Variable	N* (%)	One-Year Survival (%)	One-Year Survival (%)	Chi Square One-Year	P Value	Five-Year Survival (%)	Five-Year Survival (%)	Chi-Square Five-Year	P Value
		Yes	No			Yes	No		
Gender									
Male	2602 (40)	2056 (84.7)	382 (15.3)	173.58	<0.001	1276 (74.1)	445 (25.9)	196.94	<0.001
Female	3910 (60)	3476 (94.4)	204 (5.6)			2278 (90.3)	244 (9.7)		
Age									
<50 years	1299 (19.9)	1171 (95.4)	56 (4.6)	163.44	<0.001	801 (90.5)	84 (9.5)	121.61	<0.001
50 – 69 years	3295 (50.6)	2859 (92.7)	226 (7.3)			1823 (86.5)	285 (13.5)		
70+ years	1918 (29.5)	1502 (83.2)	304 (16.8)			930 (74.4)	320 (25.6)		
Race									
Non-Hispanic White	3606 (55.4)	3058 (89.3)	368 (10.7)	32.77	<0.001	2023 (82.6)	426 (17.4)	25.81	<0.001
Non-Hispanic Black	834 (12.8)	693 (89.4)	82 (10.6)			460 (83.2)	93 (16.8)		
Hispanic	1499 (23)	1310 (94.1)	82 (5.9)			798 (87.9)	110 (12.1)		
Asian or Pacific Islander	395 (6.1)	318 (86.9)	48 (13.1)			181 (76.0)	57 (24.0)		
American Indian or Alaska Native	61 (0.9)	51 (89.5)	6 (10.5)			35 (92.1)	3 (7.9)		
Stage at Diagnosis									
Localized	3713 (57)	3421 (98.8)	40 (1.2)	1742.98	<0.001	2178 (97.1)	66 (2.9)	1579.33	<0.001
Regional	408 (6.3)	326 (85.1)	57 (14.9)			160 (59.9)	107 (40.1)		
Distant	733 (11.3)	309 (45.7)	367 (54.3)			84 (19.8)	340 (80.2)		
Any Metastases (bone, liver, lung, and/or brain)									
None	3589 (89.8)	3133 (96.3)	119 (3.7)	1045.2	<0.001	1580 (93.2)	116 (6.8)	792.99	<0.001
Yes	407 (10.2)	167 (45.9)	197 (54.1)			28 (15.7)	150 (84.3)		
Tumor Size									
0.1cm-2.0cm	2384 (36.6)	2166 (98.5)	32 (1.5)	647.75	<0.001	1285 (96.4)	48 (3.6)	618.39	<0.001
2.1cm-5.0cm	538 (8.3)	416 (83.1)	85 (16.9)			213 (65.5)	112 (34.5)		
>5.0cm	348 (5.3)	191 (58.8)	134 (41.2)			79 (35.6)	143 (64.4)		
Surgery Status									
No or Unknown	2789 (42.8)	2122 (81.3)	488 (18.7)	437.05	<0.001	1311 (72.4)	500 (27.6)	300.33	<0.001
Yes	3723 (57.2)	3410 (97.2)	98 (2.8)			2243 (92.2)	189 (7.8)		
Radiation Therapy Status									
No or Unknown	6295 (96.7)	5395 (91.2)	523 (8.8)	114.72	<0.001	3506 (85.5)	595 (14.5)	269.59	<0.001
Yes	217 (3.3)	137 (68.5)	63 (31.5)			48 (33.8)	94 (66.2)		
Chemotherapy Status									
No or Unknown	5854 (89.9)	5167 (93.7)	350 (6.3)	678.31	<0.001	3447 (89.9)	387 (10.1)	1104.08	<0.001
Yes	658 (10.1)	365 (60.7)	236 (39.3)			107 (26.2)	302 (73.8)		
Median Household Income									
< $50,000	800 (12.3)	698 (92.6)	56 (7.4)	4.62	0.099	457 (86.4)	72 (13.6)	3.3	0.192
$50,000-$74,999	3153 (48.4)	2739 (90.1)	302 (9.9)			1838 (83.6)	360 (16.4)		
$75,000+	2558 (39.3)	2094 (90.2)	228 (9.8)			1258 (83.0)	257 (17.0)		
Population Size									
Metropolitan	5690 (87.4)	4837 (90.4)	515 (9.6)	0.09	0.764	3100 (83.6)	609 (16.4)	0.68	0.409
Non-metropolitan	821 (12.6)	694 (90.7)	71 (9.3)			453 (85.0)	80 (15.0)		

On Cox regression, patients over 70+ years of age (p<0.001, hazard ratio [HR]=2.288, 95% confidence interval [CI]=1.689-3.099) regional (p<0.001, HR=7.199, CI=4.837-10.714) and distant staging (p<0.001, HR=14.387, CI=9.608-21.541), any distant metastases (p<0.001, HR=1.349, CI=1.071-1.700) and tumor sizes >2.0cm and >5.0cm (p<0.001, HR=2.059, CI=1.466-2.891) were associated with decreased survival. On the contrary, females (p=0.003, HR=0.736, CI=0.601-0.902), Hispanic patients (p=0.013, HR= 0.711, CI=0.543-0.931), and recipients of surgery (p<0.001, HR=0.439, CI=0.347-0.556) were associated with increased survival. Full results are presented in Table 2. These findings should be interpreted in the context of known SEER limitations, including potential misclassification of cause of death and limited granularity of treatment data.

**Table 2:** Cox regression analysis for survival in patients with a GNET diagnosis. GNET: Gastric neuroendocrine tumors

Variable	P value	Hazard Ratio	95% Confidence Interval
				Gender			
Male	Reference		
Female	0.003	0.736	0.601-0.902
Age			
<50 years	Reference		
50 – 69 years	0.09	1.296	0.961-1.750
70+ years	<0.001	2.288	1.689-3.099
Race			
Non-Hispanic White	Reference		
Non-Hispanic Black	0.563	0.918	0.688-1.226
Hispanic	0.013	0.711	0.543-0.931
Asian or Pacific Islander	0.912	0.981	0.701-1.373
American Indian or Alaska Native	0.501	1.489	0.467-4.744
Stage at Diagnosis			
Localized	Reference		
Regional	<0.001	7.199	4.837-10.714
Distant	<0.001	14.387	9.608-21.541
Any Metastases (bone, liver, lung, and/or brain)			
None	Reference		
Yes	0.011	1.349	1.071-1.700
Tumor Size			
0.1cm-2.0cm	Reference		
2.1cm-5.0cm	<0.001	2.059	1.466-2.891
>5.0cm	<0.001	3.111	2.201-4.398
Surgery Status			
No or Unknown	Reference		
Yes	<0.001	0.439	0.347-0.556
Radiation Therapy Status			
No or Unknown	Reference		
Yes	0.718	0.948	0.711-1.265
Chemotherapy Status			
No or Unknown	Reference		
Yes	0.103	1.202	0.963-1.499
Median household income			
< $50,000	Reference		
$50-74,999	0.761	1.058	0.738-1.515
$75,000+	0.328	0.822	0.556-1.217
Urban-Rural Continuum			
Metropolitan	Reference		
Non-Metropolitan	0.585	0.906	0.635-1.292

## Discussion

This study provides a comprehensive analysis of the epidemiology and prognosis of gastric neuroendocrine tumors (GNETs) in a large, population-based cohort. Among 6,512 patients, the overall outcomes were favorable with one- and five-year CSS rates of 90.4% and 83.8%, respectively. The incidence of GNETs increased significantly by 104.1% between 2000 and 2020, with an average annual percent change of 4.27%. Prognostic analysis revealed that female sex, Hispanic ethnicity, and surgical intervention were associated with improved survival, whereas age ≥70 years, larger tumor size (>2.0cm and >5.0cm), regional or distant stage, and presence of metastases predicted worse survival outcomes.

Our analysis confirmed staging as one of the strongest prognostic factors for GNETs, reinforcing its central role in predicting patient outcomes. Compared with localized GNETs, regional and distant disease conferred a 7.2-fold and 14.4-fold increased risk of mortality, respectively. These stage-stratified risks mirror prior SEER-based studies demonstrating the unfavorable impact of advanced stage in GNETs [3,4], and are consistent with broader gastroenteropancreatic NET (GEP-NET) analyses showing more than a 10-fold increase in mortality for distant versus localized tumors [11]. Survival differences by stage are striking, with 5-year survival exceeding 90% in localized GNETs [11], declining to 53% in regional nodal disease [11], and dropping to approximately 5% in poorly differentiated gastric neuroendocrine tumors [15]. Together, our results add to the growing body of literature reinforcing the importance of stage at diagnosis as a critical determinant of prognosis and a guide for treatment decisions.

Tumor size was also a significant determinant of survival in our cohort. Our results confirm those of prior studies that conclude increasing tumor size is associated with adverse outcomes [3,4]. However, our findings differ on the inflection threshold. While earlier reports have suggested tumor size >1 cm serving as an independent predictor of poor outcome, including tumor-related death, metastases, or angioinvasion [16], our analysis demonstrated that survival risk was more accurately stratified at higher thresholds. Tumors >2cm were associated with a two-fold increased risk of mortality, while tumors >5cm carried more than a three-fold risk compared to lesions ≤2cm. Biologically, tumor size likely reflects both tumor aggressiveness and the likelihood of regional or distant spread, which may explain its association with poorer survival [9,11]. These findings underscore the clinical value of tumor size in GNETs and suggest that thresholds >2cm may provide a more meaningful cutoff for risk stratification, surveillance, and treatment planning.

Alongside prognostic determinants, our study demonstrates a persistent rise in GNET incidence, aligning with prior population-based and international reports which have documented steadily increasing rates over recent decades [3-8]. Earlier SEER analyses demonstrated an increase from 0.031 to 0.485 cases per 100,000 individuals [3], with subsequent updates reporting a further rise to 0.615 per 100,000 [4]. In our cohort, incidence continued to climb, reaching a peak of 0.680 per 100,000, representing a 104% overall increase for included dates and an average annual percent increase of approximately 4.3%. The rising incidence of GNETs is likely multifactorial and reflects both enhanced detection and evolving population risks. Greater use of gastrointestinal endoscopy has been repeatedly identified as a major contributor, with advances and increased utilization facilitating earlier recognition of localized disease [17-19]. The broader application of immunohistochemistry has also been pivotal, as routine staining with neuroendocrine markers such as synaptophysin and chromogranin A enables accurate classification and prevents a substantial number of tumors from being overlooked [18,20]. Improved registry capture has further amplified reported case numbers, as demonstrated in population-based analyses from SEER that highlight the effect of histologic confirmation and more complete data abstraction on incidence estimates [5,11,21]. Finally, changes in population risk exposures may contribute, with chronic hypergastrinemia from autoimmune gastritis, pernicious anemia, long-term proton pump inhibitor use, and Zollinger-Ellison syndrome all recognized as predisposing conditions [22,23]. Additional associations include comorbidities such as diabetes, hypertension, and dyslipidemia, as well as Helicobacter pylori infections [24]. Collectively, the sustained rise in GNET incidence likely reflects a combination of improved endoscopic and pathological detection, enhanced registry capture and histologic classification within SEER, and evolving population risk factors. These trends are best clarified through future studies that integrate detailed clinical datasets with administrative and registry-based data sources.

The unadjusted chi-square analyses demonstrated higher one- and five-year cancer-specific survival among patients who did not receive chemotherapy or radiation. However, these findings likely reflect confounding by indication, as patients receiving systemic therapy or radiation are more likely to have advanced-stage or metastatic disease. Therefore, these bivariate comparisons should not be interpreted as evidence that treatment worsens survival. In contrast, multivariable Cox regression, adjusting for stage, tumor size, and metastasis, demonstrated that receipt of surgery was independently associated with improved survival, whereas chemotherapy and radiation were not independently significant predictors.

Outcomes among GNET patients were influenced by multiple demographic variables in our study. Younger patients (<50 years) experienced improved survival, while those ≥70 years had significantly worse outcomes. These results align with prior studies identifying age ≥65 years as an independent predictor of overall and disease-specific survival, with risk differences persisting over time [25]. To our knowledge, this is the first study to demonstrate a female survival advantage in GNETs. Although sex-based differences have been described in broader GEP-NET populations [26,27], their relevance in GNETs has not been previously established. Notably, our analysis suggests female sex and Hispanic ethnicity as novel demographic factors associated with longer survival in GNETs, highlighting areas for further investigation into potential biological, socioeconomic, and healthcare access determinants of outcomes. Potential explanations include differences in lifestyle exposures, such as tobacco and alcohol use, and the influence of estrogen on tumor biology through effects on cell proliferation, apoptosis, and immune response [27]. We also observed improved survival among Hispanic patients, representing another novel contribution to the GNET literature. These findings highlight the relevance of clinical and demographic factors in prognostic assessment and support future research into survival disparities.

While these findings provide important contributions to the understanding of GNET epidemiology and prognosis, several limitations must be acknowledged. Despite using a large cancer registry, the overall sample size of n=6,512 is still relatively small. The retrospective nature of the study leads to conclusions being drawn based on associations rather than causational patterns better attained from prospective studies. This analysis also relied on the SEER database, which is subject to miscoding, missing variables, heterogeneity in reporting, and incomplete case-level data capture inherent to registry-based datasets [12]. As with all routinely collected administrative datasets, SEER is vulnerable to misclassification bias and temporal changes in coding practices that may influence variable definitions over time. Variables coded as missing or “unknown” may reflect incomplete abstraction rather than the true absence of treatment or disease characteristics. As a result, some variables may be missing or unknown and certain covariates may be underreported. Because SEER captures a large, geographically diverse segment of the U.S. population, these findings are broadly generalizable to U.S. patients with GNET; however, applicability to healthcare systems with different referral patterns, access to care, or ethnic compositions may be limited. Additionally, important clinical details such as patient comorbidities, insurance status, Ki-67 index, surgical margins, and systemic therapy regimens were unavailable, limiting risk adjustment. The absence of these variables introduces the possibility of residual confounding, particularly in the interpretation of demographic and treatment-associated survival differences. Accordingly, these associations should not be interpreted as evidence of independent causal effects. Stage, metastasis, and tumor size are clinically related variables and may exhibit correlation. Although each represents a distinct dimension of tumor burden, some degree of collinearity may influence effect estimates and should be considered when interpreting hazard ratios. Treatment variables lacked information on surgical intent, margin status, and systemic therapy sequencing, which may influence outcomes. Finally, although this is one of the largest cohorts of GNET patients studied to date, subgroup analyses, particularly by race, were constrained by small sample sizes, limiting statistical power to detect smaller group differences.

## Conclusions

In this large, population-based analysis of 6,512 patients, we demonstrate that GNET incidence has continued to rise steadily over the past two decades and that survival is strongly influenced by clinical and demographic factors. Advanced age, larger tumor size, regional or distant stage, and metastatic disease were independently associated with worse outcomes, whereas female sex, Hispanic ethnicity, and receipt of surgery were associated with improved survival. These findings reinforce the prognostic importance of stage and tumor burden, highlight demographic differences that warrant further study, and offer updated incidence trends that reflect both growing recognition of GNETs and potential shifts in underlying risk exposures. Given that these findings are derived from a large population-based registry, they highlight important associations for clinical consideration but warrant validation in prospective cohorts with more granular clinical data to address potential confounding and misclassification inherent in administrative datasets. As the incidence of GNETs continues to increase, a better understanding of these risk factors is essential for improving early detection, refining risk stratification, and guiding evidence-based management. Future studies integrating detailed treatment records, comorbidity data, and tumor biomarkers such as Ki-67 with population registries are essential to refine prognostic models, clarify mechanisms underlying the observed demographic disparities, and distinguish improved detection from true changes in disease burden, ultimately helping to further improve outcomes for patients with GNETs.
